# Genomic heterozygosity and hybrid breakdown in cotton (*Gossypium*): different traits, different effects

**DOI:** 10.1186/s12863-016-0366-5

**Published:** 2016-04-12

**Authors:** Baosheng Dai, Huanle Guo, Cong Huang, Xianlong Zhang, Zhongxu Lin

**Affiliations:** National Key Laboratory of Crop Genetic Improvement, Huazhong Agricultural University, Wuhan, 430070 Hubei China

**Keywords:** Genomic heterozygosity, Hybrid breakdown, *Gossypium*, Linkage disequilibrium

## Abstract

**Background:**

Hybrid breakdown has been well documented in various species. Relationships between genomic heterozygosity and traits-fitness have been extensively explored especially in the natural populations. But correlations between genomic heterozygosity and vegetative and reproductive traits in cotton interspecific populations have not been studied. In the current study, two reciprocal F_2_ populations were developed using *Gossypium hirsutum* cv. Emian 22 and *G. barbadense* acc. 3*–*79 as parents to study hybrid breakdown in cotton*.* A total of 125 simple sequence repeat (SSR) markers were used to genotype the two F_2_ interspecific populations.

**Results:**

To guarantee mutual independence among the genotyped markers, the 125 SSR markers were checked by the linkage disequilibrium analysis. To our knowledge, this is a novel approach to evaluate the individual genomic heterozygosity. After marker checking, 83 common loci were used to assess the extent of genomic heterozygosity. Hybrid breakdown was found extensively in the two interspecific F_2_ populations particularly on the reproductive traits because of the infertility and the bare seeds. And then, the relationships between the genomic heterozygosity and the vegetative reproductive traits were investigated. The only relationships between hybrid breakdown and heterozygosity were observed in the (Emian22 × 3–79) F_2_ population for seed index (SI) and boll number per plant (BN). The maternal cytoplasmic environment may have a significant effect on genomic heterozygosity and on correlations between heterozygosity and reproductive traits.

**Conclusions:**

A novel approach was used to evaluate genomic heterozygosity in cotton; and hybrid breakdown was observed in reproductive traits in cotton. These findings may offer new insight into hybrid breakdown in allotetraploid cotton interspecific hybrids, and may be useful for the development of interspecific hybrids for cotton genetic improvement.

**Electronic supplementary material:**

The online version of this article (doi:10.1186/s12863-016-0366-5) contains supplementary material, which is available to authorized users.

## Background

Species divergence is currently an area of intense study [1, 2]. One related topic that has been received an increasing amount of attention is the examination of phenotypes expressed by hybrids between species [3]. Hybrid breakdown is the loss of fitness in hybrids between species, showing as inferior viability and fertility in F_2_ and later generations [3]. Hybrid breakdown can be viewed as an indicator of an early stage in the evolution of a new species, so this phenomenon may provide clues into the genetics of speciation [4]. Hybrid breakdown has been well documented in both plant and animal hybrids, such as in the inter-subspecific *O. sativa* ssp. japonica × ssp. indica hybrid [5, 6], and the hybrid parasitoid wasp genus *Nasonia* [7]. Heterozygosity–fitness correlations have been used to study the relationships between genomic heterozygosity and fitness-related traits at the individual level in a variety of organisms [8–12].

Several alternative genetic explanations for the prevalence of hybrid breakdown have been reported in recent studies including Bateson–Dobzhansky–Muller (BDM) incompatibilities [13], the incompatibilities between the nuclear genome and the organellar genomes of mitochondria and chloroplasts [4], and disruption of co-adapted gene complexes [14]. The BDM model of incompatibility involves a deleterious epistatic interaction between alleles at two different loci affecting the descendant of the interspecific hybrid as much as an inter-subspecific hybrid. Several pairs of epistatic alleles are responsible for hybrid breakdown between japonica and indica cultivars of rice, which have been mapped to specific genomic regions [3, 5, 15]. The hybrid breakdown of *Arabidopsis* hybrids has been ascribed to BDM incompatibility involving reciprocal silencing of duplicated genes [16].

The molecular mechanisms of hybrid breakdown underlying nucleo-cytoplasmic genomic interactions have been well demonstrated [4]. Given the co-evolution of the organellar genomes and the nuclear genome, the disruption of inter-genomic coadaptation can result in organelle dysfunction and consequent hybrid breakdown. The fitness loss in marine copepod *Tigriopus californicus* hybrids is completely attributable to nuclear–mitochondrial genomic interactions which led to reduced ATP synthesis [17]; the nuclear–cytoplasmic data revealed an increased tendency towards maladaptation in inter-population crosses [14].

The theory of co-adapted gene complexes suggests that gene combinations are co-adapted if high fitness depends on specific interactions between them; such gene combinations are referred to as co-adapted gene complexes [18]. A consequent loss of heterosis in *Drosophila* hybrid populations was ascribed to breakdown of co-adapted gene complexes [19].

Although several theories have been used to explain the genetic causes underlying hybrid breakdown, heterozygosity–fitness correlations have rarely been studied in crop plants. Nevertheless, various evolutionary biology studies have examined relationships between individual genomic heterozygosity and fitness using heterozygosity–fitness correlations [20–24]. Individual genomic heterozygosity is usually estimated using neutral genetic markers, such as simple sequence repeats (SSRs) or single-nucleotide polymorphisms (SNPs). Two hypotheses have been used to explain heterozygosity–fitness correlations in interspecific hybrids: outbreeding depression [25] and local effects caused by functional genes neighbouring neutral markers, which led to the observed correlations [26].

Relationships between genomic heterozygosity and trait-fitness have been extensively explored because they have strong implications for ecology and evolution. Heterozygosity–fitness correlations have been used to study relationships between genomic heterozygosity and fitness-related traits at the individual level in natural hybrid populations of a variety of organisms [8–12]. Assessment of individual genomic heterozygosity has a very important role in determining heterozygosity–fitness correlations. Several studies have utilized a molecular hybrid index (MHI) to measure genomic heterozygosity [24, 27, 28]. To increase the accuracy when calculating genomic heterozygosity, the effects of marker number and marker type have been studied [24, 27, 29]. Miller et al. [27] argued that SNPs performed similarly to microsatellites in terms of precision and accuracy in genomic heterozygosity calculations.

The genus *Gossypium* includes four important cultivated species, *G. hirsutum*, *G. barbadense*, *G. arboreum* and *G. herbaceum* [30]. Each species has unique advantageous traits that are imperative for cotton breeding. To combine the advantages of each, interspecific crossings between *G. hirsutum* and *G. barbadense* have been performed extensively [31]. The cross between the allotetraploid cottons is straightforward and can produce vigorous fertile F_1_ hybrids; but serious segregation occurs in later generations, which contain many weak, and even infertile plants [32, 33], known as hybrid breakdown. Stephens found that selective elimination of a donor parent genotype, detected in interspecific backcrosses involving *G. hirsutum* and *G. barbadense*, was the likely cause of the breakdown [33].

Correlations between genomic heterozygosity and vegetative and reproductive traits in cotton interspecific populations have not been studied. In the current study, we developed reciprocal F_2_ populations derived from the cross between *G. hirsutum* cv. Emian 22 (denoted as E22) and *G. barbadense* acc. 3*–*79 (denoted as 3–79), including a direct cross (E22 × 3–79) F_2_ population (denoted as (E3) F_2_) and a reciprocal cross (3–79 × E22) F_2_ population (denoted as (3E) F_2_). This mating design allowed us to investigate maternal effects. Here, we first present an improved approach to evaluate individual genomic heterozygosity. Second, we investigate the distribution of individual genomic heterozygosity in interspecific F_2_ populations of allotetraploid cottons. Third, we investigate hybrid breakdown of vegetative and reproductive traits in reciprocal F_2_ populations. We also investigate the relationships between genomic heterozygosity and vegetative and reproductive traits.

## Methods

### Plant materials

The plant materials, *G. hirsutum* cv. Emian 22 (E22) and *G. barbadens*e acc. 3–79 were collected for scientific research from Huanggang Academy of Agricultural Sciences (HGAAS, Huanggang, China) and the Institute of Cotton Research, Chinese Academy of Agricultural Sciences (CAAS, Anyang, China), respectively. E22 is an elite upland cotton cultivar developed by HGAAS and available in China; it was approved by the National Crops Variety Approval Committee (China) in 2000, and the authorized number was “Guoshenmian 20000006”. *G. barbadens*e acc. 3–79 is the genetic and cytogenetic standard line of *G. barbadens*e and worldwidely available; it was identified by Kohel RJ [34]. The two cotton materials are not the specimens deposited in a herbarium and deposited in the Group of Cotton Genetic Improvement (GCGI) of National Key Laboratory of Crop Genetic Improvement (China).

E22 and 3–79 were used as parents to develop F_2_ segregating populations. To investigate maternal effect, we constructed an F_2_ population with 142 individuals from an E22 (female parent) × 3–79 (male parent) direct cross, as well as an F_2_ population with 142 individuals from a 3–79 (female parent) × E22 (male parent) reciprocal cross. The benefit of this experimental design is that distributions of individual genomic heterozygosity can be observed in two different maternal cytoplasmic backgrounds under the same genomic background. This strategy allows us to distinguish effects from different maternal cytoplasmic backgrounds on the relationships between genomic heterozygosity and vegetative and reproductive traits.

### Phenotyping vegetative and reproductive traits

The reciprocal F_2_ populations together with their parents, E22 and 3–79, were grown and evaluated in the cotton breeding station at Huazhong Agricultural University, Wuhan, China in 2013. Space between the plants was maintained at 35 cm within the rows and at 100 cm between rows, and plant density was maintained at approximate 27 500 plants ha^−1^.

To investigate hybrid breakdown in these interspecific crossing populations, two plant vegetative traits: plant height (PH) and branch number (BrN)) and three reproductive traits: boll number (BN), seed set weight(SW) and seed index (SI) were chosen according to the life history of cultivated cotton. Since cultivated cotton is annual crops, the plant height and branch number in the boll-forming stages could generally reflect the vegetative viability; the boll number, seed set weight and seed index could generally reflect the reproductive fertility. Two plant vegetative traits, PH and BrN, were investigated on August 15 before pinching. Total BN was explored on September 15 before the approach of early frost. For each plant, SW was determined by weight of the total seed after the cotton ginning processing. To evaluate seed vigor and plumpness, the SI was evaluated for every plant. Descriptive statistical parameters were obtained for each trait.

To assess the relationship between different traits in the reciprocal F_2_ populations, Pearson’s correlation coefficients were calculated for traits of individual plants in each population.

### Molecular marker genotyping

Total genomic DNA of the parents and individuals of the two F_2_ segregating populations was extracted from young leaves according to procedures described by Paterson et al. [35]. Since mutual independence is latent premise for MHI calculating, a total of 125 SSRs were randomly selected from the 2316 genome-wide loci according to the genetic distance in the interspecific genetic map previously constructed based on the same parents [36]. These SSR markers were used to genotype the two interspecific reciprocal F_2_ populations. The detail of these markers was presented in Additional file 1: Table S1. The sequences of molecular markers can be obtained from CottonGen (http://www.cottongen.org) [37]. Polymerase chain reaction (PCR) analysis, electrophoresis and silver staining were performed according to procedures described by Lin et al. [38].

### Linkage disequilibrium analysis

Correlations between genotyped markers could lead to deviations in the estimated value of individual molecular hybrid index (MHI). To avoid the correlation, independent assortment between the loci must be validated by Linkage disequilibrium (LD) analysis. LD analysis between all pairs of loci was implemented using the software package TASSEL, which was developed by Edward Buckler’s group [39]. LD was estimated by squared allele-frequency correlations (r^2^). According to the definition of LD and calculation formula for r^2^, the bigger r^2^ value is, the degree of correlations or linkage is closer. Correlation was considered to be significant if r^2^ > 0.2, and one marker from these pairs was removed. In the end, all markers with r^2^ < 0.2 were used to estimate individual genomic heterozygosity. After LD analysis, to investigate the correlation between these studied traits and these remaining SSR loci, QTL for these studied traits were searched in these loci from the Cotton QTL Database (http://www2.cottonqtldb.org:8081/index) [40].

### Measurement of genomic heterozygosity

MHI is an estimate of the proportion of alleles based on molecular markers inherited from alternative parental species in hybrid population [41]. For the interspecific hybrid population from two species, one species is designated as the reference species, the other as the alternative. MHI values range from 0 to 1, corresponding to pure individuals of alternative and reference species, respectively [41]. MHI is calculated as a measure of individual genomic heterozygosity. Assessment of MHI was performed using the *est. h* function incorporated in the R program INTROGRESS [42]. This function renders a maximum likelihood hybrid index estimate for each potentially admixed individual and a 95 % confidence interval for each [42, 43].

### Analysis of the effects of genomic heterozygosity effect on plant traits

To assess correlations between genomic heterozygosity and plant traits, Pearson’s correlation analysis and one-way analysis of variance (ANOVA) were used to evaluate both populations included in this study.

First, due to the nature of the MHI, the average value for most heterozygous individuals is 0.5. Therefore, we divided every population into two groups based on the MHI value of each individual: the members in the first group possessed MHI values greater than 0.5, and those in the other group had values less than 0.5. Subsequently, Pearson’s correlation coefficients were calculated between each trait and the individual heterozygosity value of each plant in every group.

Second, to discover trait differentiation at different genomic heterozygosity levels, the individuals in the two populations were divided into six groups in accordance with individual MHI values, with a step size of 0.05. The average of the studied traits of each level was calculated for each population, and the statistical significance among groups for each trait was determined by performing separate one-way ANOVAs. Multiple comparisons between each level of genomic heterozygosity were performed using Tukey’s multiple comparison tests.

## Results

### Hybrid breakdown in interspecific reciprocal F_2_ populations

**Fig. 1:**
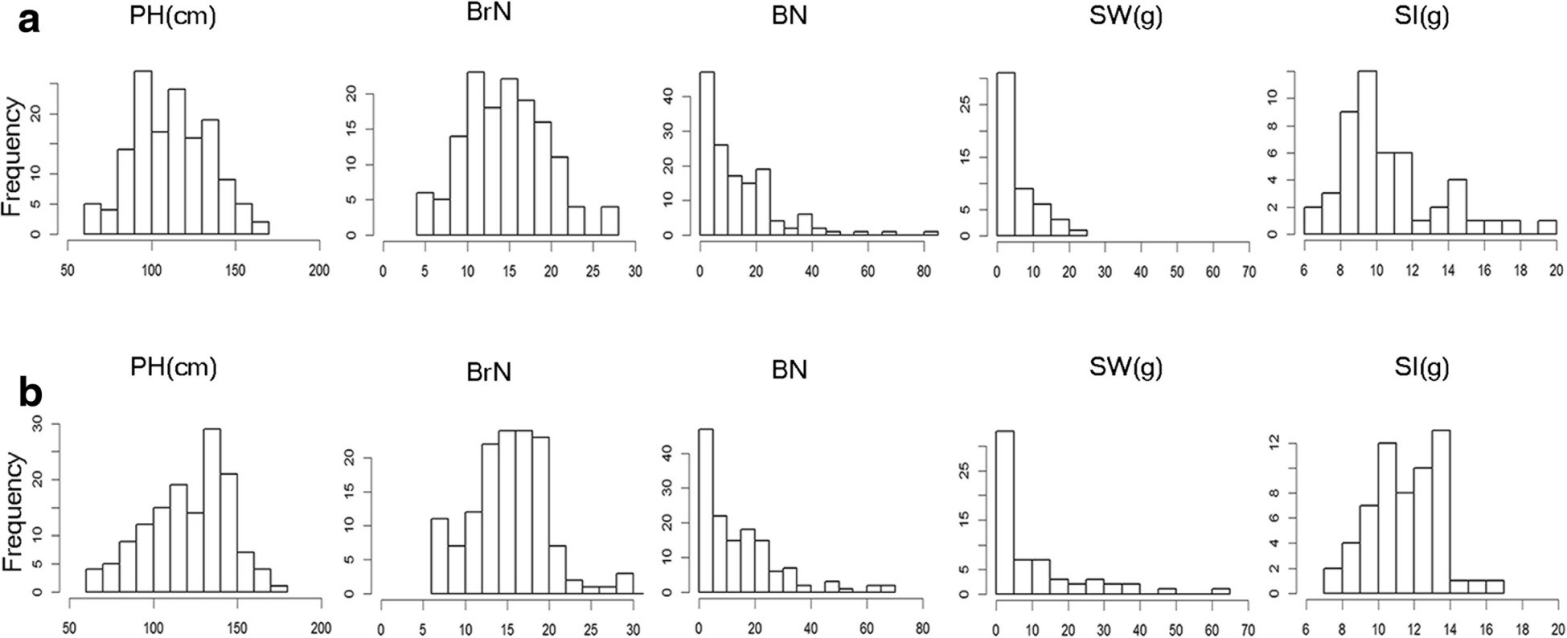
Distribution of plant traits in the two evaluated F_2_ populations. **a**: (3E) F_2,_ **b**: (E3) F_2._ PH, BrN, BN, SW and SI are abbreviated for plant height, branch number, boll number, seed set weight (g), seed index (g), respectively

In total, 142 plants were genotyped simultaneously in each of two interspecific reciprocal F_2_ populations. Although two plants died after DNA sampling in the (E3) F_2_ population, the remaining 140 individuals were phenotyped. PH, BN and BrN were investigated for each plant in these two populations. Owing to sterility or flowering delay, only 62 individuals in the (E3) F_2_ population and 51 in the (3E) F_2_ population were surveyed for two reproductive traits, SW and SI. Frequency distributions of the two interspecific F_2_ populations for PH, BrN, BN, SW and SI are presented in Fig. 1. Descriptive statistical parameters, such as the mean value, standard deviation, range, skewness, kurtosis and probability of normal distribution for each trait, are shown in Table 1. For both populations, only two traits displayed a normal distribution, PH (*p* = 0.20) in (3E) F_2_ and SI (*p* = 0.69) in (E3) F_2_. The remaining traits displayed non-normal distributions. The data demonstrated that hybrid breakdown may exist in the interspecific reciprocal F_2_ populations particularly with respect to reproductive traits such as infertility and bare seeds.

**Table 1:** Descriptive statistics of plant traits in the two evaluated F_2_ populations

Population	Trait	Size	Range	Mean ± S.D.	SKE.	KUR	NT.W	NT.p
(3E) F_2_	Plant height (cm)	142	60.00–165.00	114.32 ± 22.21	0.05	−0.48	0.99	0.20
	Branch number	142	5.00–28.00	15.06 ± 4.88	0.25	−0.24	0.98	0.12
	Boll number	142	0.00–81.00	14.16 ± 13.66	1.78	4.55	0.84	0.00
	Seed set weight (g)	51	0.32–20.77	5.86 ± 5.15	1.26	0.52	0.83	0.00
	Seed index (g)	50	6.10–19.58	10.75 ± 2.86	1.12	0.82	0.90	0.00
(E3) F_2_	Plant height (cm)	140	60.00–180.00	123.75 ± 25.18	−0.35	−0.49	0.98	0.02
	Branch number	138	6.00–38.00	15.95 ± 5.01	0.77	2.35	0.95	0.00
	Boll number	140	0.00–69.00	14.82 ± 14.74	1.50	2.52	0.85	0.00
	Seed set weight (g)	62	0.32–63.44	10.47 ± 13.23	1.87	3.32	0.74	0.00
	Seed index (g)	60	7.14–16.64	11.47 ± 1.96	0.03	−0.33	0.99	0.69

To investigate possible maternal effects on hybrid breakdown, a two-sample Kolmogorov–Smirnov test was performed between the two populations for the five plant traits. Statistical significance was observed for PH (*D* = 0.2173, *p* = 0.0026) and SI (*D* = 0.3577, *p* = 0.0021), suggesting that the maternal cytoplasmic environment could have significant effects on PH and SI.

Correlations between the vegetative and reproductive traits in the two interspecific reciprocal F_2_ populations are listed in Table 2. In the (E3) F_2_ population, significantly positive correlations were observed between PH and BrN (*r* = 0.42, *p* = 0) as well as between BN and SW (*r* = 0.40, *p* = 0). In the (3E) F_2_ population, significantly positive correlations were observed between PH and SW (*r* = 0.28, *p* = 0.04), PH and BN (*r* = 0.38, *p* = 0), PH and BrN (*r* = 0.37, *p* = 0), BN and BrN (*r* = 0.34, *p* = 0), and BN and SW (*r* = 0.64, *p* = 0). These data implied that the correlations among the vegetative and reproductive traits in the (E3) F_2_ population were non-significant; however, this was not the case in the (3E) F_2_ population.

**Table 2:** Pearson’s correlation coefficients between plant traits in the two evaluated populations

	Plant height (cm)	Branch number	Boll number	Seed set weight (g)	Seed index (g)
Plant height (cm)	–	0.42**	0.12	0.14	0.16
Branch number	0.37**	–	0.15	0.05	−0.13
Boll number	0.38**	0.34**	–	0.40**	−0.07
Seed set weight (g)	0.28*	0.27	0.64**	–	0.11
Seed index (g)	−0.03	−0.03	−0.03	0.07	–

Entries above the diagonal represent the correlation coefficient for (E3) F_2_; entries below the diagonal represent the correlation coefficient for (3E) F_2_. **p < 0.01,*p < 0.05

### Loci independence check by linkage disequilibrium analysis

**Fig. 2:**
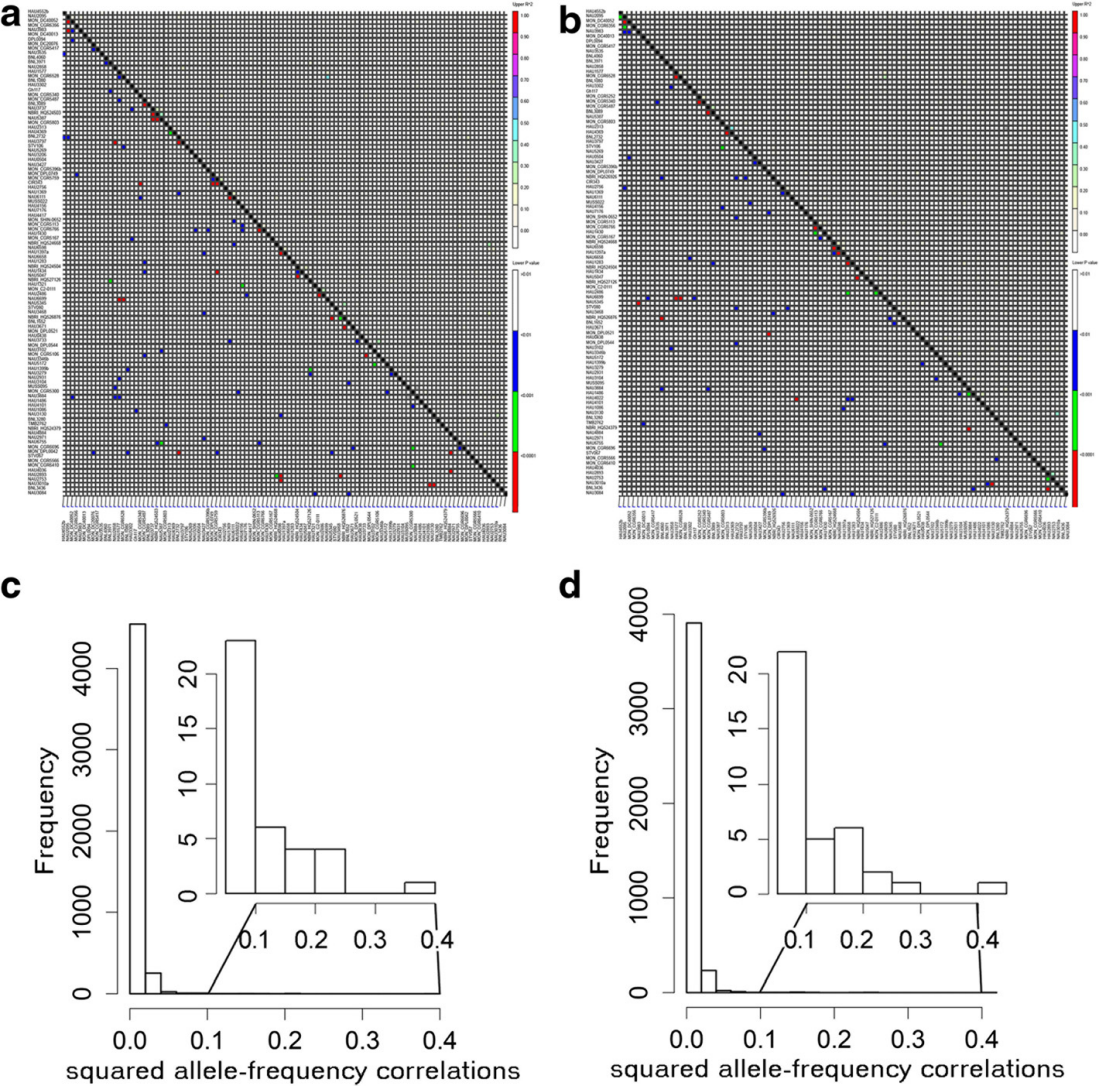
Pairwise linkage disequilibrium between all markers in both populations. The r^2^ and p-values from linkage disequilibrium testing of the (3E) F_2_ (**a**) and (E3) F_2_ (**b**) are above the diagonal and below the diagonal, respectively. The r^2^ distributions in (3E) F_2_ (**c**) and (E3) F_2_ (**d**) are shown

To avoid the correlation between paired loci, mutual independence between the loci must be validated by LD analysis. The r^2^ and p values between all loci pairs based on LD testing and the r^2^ distributions of the two F_2_ populations are presented in Fig. 2. After pre-processing, 87 loci were shared in the two interspecific reciprocal F_2_ populations. Four locus pairs showed significant LD. Following the above, one marker from each locus pair was removed from the dataset. To test the substitution effect produced by this method, molecular hybrid indices for each marker from these pairs were calculated (Additional file 2: Tables S2 and Additional file 3: Table S3). To determine the statistical significance of the differences among these MHIs, a two-sample Kolmogorov–Smirnov test was performed between each in the two populations. The results showed that no statistical significance was observed between MHIs after replacement in the (3E) F_2_ population and (E3) F_2_ population. That is, if NAU3130 was replaced with NAU3010a (*D* = 0.0704, *p* = 0.87), NAU5345 was replaced with NBRI_HQ526876 (*D* = 0.0845, *p* = 0.69), or the two markers were replaced together (*D* = 0.0845, *p* = 0.69) in the (3E) F_2_ population, there was no statistically significant difference. Similarly, in the (E3) F_2_ population, if HAU2313 was replaced with HAU4369 (*D* = 0.0786, *p* = 0.78), HAU1577 was replaced with MON-CGR6528 (*D* = 0.0571, *p* = 0.97) or the two markers were replaced together (*D* = 0.0571, *p* = 0.97), there was no significant difference. These results indicated that this method was in accordance with our expectations. Meanwhile, to investigate correlation between the studied traits and these remaining SSR loci, QTL correlated to the studied traits were searched from Cotton QTL Database (http://www2.cottonqtldb.org:8081/index) [40], the results (Additional file 4: Tables S4) showed no markers were correlated to the studied traits. The remaining 83 neutral loci in the two populations were used for further analysis.

### Distribution of individual genomic heterozygosity

**Fig. 3:**
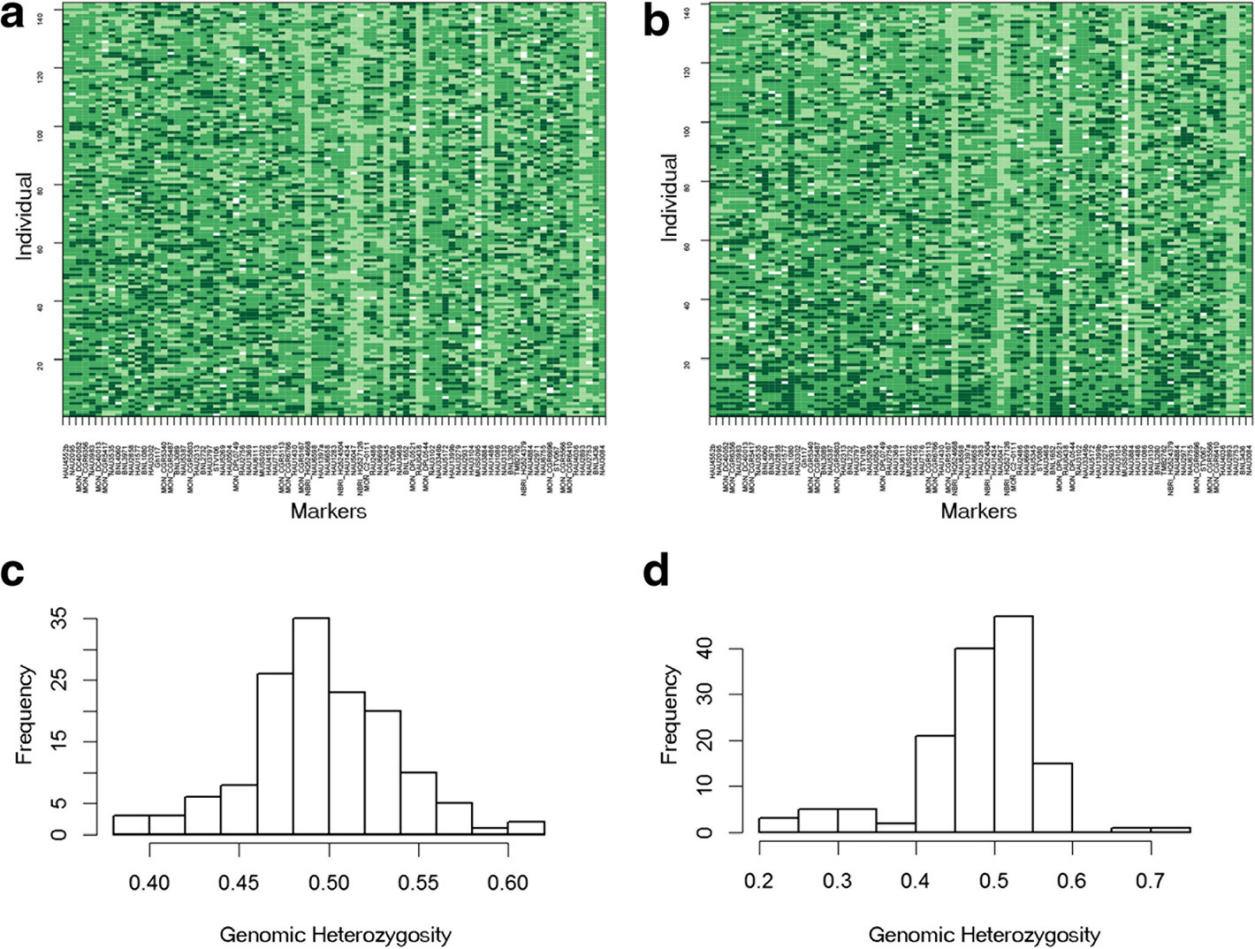
The constructed specifications for all markers and individuals in (3E) F_2_ (**a**) and (E3) F_2_ (**b**) and the frequency distributions of MHIs in (3E) F_2_ (**c**) and (E3) F_2_ (**d**). Each rectangle denotes an individual’s genotype at a given locus. Colours are arranged from darker green, indicating E22 homozygotes, to green, indicating heterozygote genotypes between E22 and 3–79, to light green, indicating 3–79 homozygotes. White blocks indicate missing data

Individual genomic composition patterns were found in the 83 SSR loci, as shown in Fig. 3 (a, b). Allele distribution from E22 and 3–79 occurred randomly both among the individuals in the two populations and among different genomic regions in individuals.

Individual genomic heterozygosity was estimated using the 83 SSR loci common to the two populations. Frequency distributions of the MHIs in the two interspecific reciprocal F_2_ populations are presented in Fig 3 (c, d).

In the (3E) F_2_ population, the MHI ranged from 0.39 to 0.62 with a mean and standard deviation of 0.497 and 0.040, respectively. The Shapiro-Wilk test revealed that the distribution of the MHI was normal (*W* = 0.9906, *p* = 0.4645). In the (E3) F_2_ population, the MHI ranged from 0.21 to 0.71 with a mean and standard deviation of 0.480 and 0.080, respectively. The MHI distribution was assessed for normality using the Shapiro-Wilk test, and an abnormal distribution was noted (*W* = 0.8988, *p* = 2.713e-08).

The average MHI in each population was less than the expected value (0.5), indicating that the genomic composition from 3–79 was greater than that from E22, which suggests that the alleles from 3–79 may have a stronger selective advantage.

To investigate the statistical significance of the difference in the MHIs, a two-sample Kolmogorov–Smirnov test was performed on the two populations, which indicated that the two distributions were not drawn randomly from the same population (D = 0.1732, *p* = 0.0290). This result suggests that the maternal cytoplasmic environment may have significant effects on individual genomic heterozygosity.

### Relationship between genomic heterozygosity and hybrid breakdown

Pearson’s correlation analysis and one-way ANOVA were performed to detect the relationship between genomic heterozygosity and hybrid breakdown. Two groups were divided from each population according to individual MHIs; one group had an MHI greater than 0.5, and the other group did not.

The resulting Pearson’s correlation coefficients in the two populations are presented in Table 3. Significant correlations between MHI and BN (*r* = 0.54, *p* = 0) as well as between MHI and SW (*r* = 0.70, *p* = 0) were observed in the group (MHI < 0.5) from the (E3) F_2_ population. However, the other *p*-values of the Pearson’s correlation coefficients were greater than 0.05, demonstrating that no significant correlations were found. In other words, hybrid breakdown relative to the other traits in the interspecific reciprocal F_2_ populations was unrelated to genomic heterozygosity.

**Table 3:** Pearson’s correlation coefficients between plant traits and MHI in both populations

Correlation	(3E) F_2_				(E3) F_2_			
	MHI < 0.5		MHI > 0.5		MHI < 0.5		MHI > 0.5	
	*r*	*p*	*r*	*p*	*r*	*p*	*R*	*p*
Plant height (cm)	−0.12	1	0.09	1	−0.09	1	0.11	1
Branch number	−0.07	1	0.01	1	−0.13	1	0.2	1
Boll number	0.05	1	0.02	1	−0.54	0	0.18	1
Seed set weight (g)	−0.12	1	0.13	1	−0.7	0	0.17	1
Seed index (g)	0.37	0.83	−0.09	1	−0.2	1	−0.03	1

According to individual MHIs, six groups were defined in the two populations, with levels 1 to level 6 including all observed values. The number of individuals and the mean and MHI range of each of the six levels are presented in Table 4. These results are consistent with the expectations of the F_2_ population, namely, 62.14 % of individuals in the (E3) F_2_ population and 80.28 % of individuals in the (3E) F_2_ population were noted in levels 3 and 4, with MHIs ranging from 0.45 to 0.55.

**Table 4:** MHI range, mean and observation number (%) for the six genomic heterozygosity levels in the two evaluated populations

Level	(3E) F_2_			(E3) F_2_		
	Size	Mean	Percentage (%)	Size	Mean	Percentage (%)
<0.4	3	0.40	2.11	15	0.30	10.71
0.4 ~ 0.45	12	0.43	8.45	21	0.43	15.00
0.45 ~ 0.5	62	0.48	43.66	37	0.48	26.43
0.5 ~ 0.55	52	0.52	36.62	50	0.52	35.71
0.55 ~ 0.6	11	0.56	7.75	15	0.57	10.71
>0.6	2	0.61	1.41	2	0.69	1.43

The mean trait performance at each level of genomic heterozygosity is presented in Table 5. One-way ANOVA was used to determine group differences between these two populations. No significant differences were observed between plant traits and individual genomic heterozygosity levels in the (3E) F_2_ population. Nevertheless, significant differences were observed between BN (*F*_5, 134_ = 4.925, *p* = 0.00036) and SW (*F*_5, 55_ = 10.59, *p* = 3.72e-07) and individual genomic heterozygosity level in the (E3) F_2_ population. These results reconfirmed that plant vegetative traits, i.e., PH and BrN, have no correlation with individual genomic heterozygosity; however, genomic heterozygosity may affect reproductive traits, such as BN and SW.

**Table 5:** Comparisons across MHI levels in trait performances from both populations

Level	(3E) F_2_					(E3) F_2_				
	PH(cm)	BrN	BN	SW(g)	SI(g)	PH(cm)	BrN	BN	SW(g)	SI(g)
<0.4	108.00	13.33	4.33	1.24	6.10	118.60	16.47	29.80 b	29.73 b	11.93
0.4 ~ 0.45	118.25	15.83	12.25	9.39	8.86	123.00	16.71	14.24 a	6.62 a	11.13
0.45 ~ 0.5	109.50	14.37	12.27	6.08	10.09	116.95	15.58	9.05 a	3.61 a	11.18
0.5 ~ 0.55	117.35	15.77	16.38	5.35	11.59	129.52	15.59	14.36 a	6.86 a	11.69
0.55 ~ 0.6	127.73	16.27	20.73	6.97	10.93	125.67	16.07	15.67 ab	8.18 a	11.14
>0.6	97.00	9.50	5.00	–	–	137.50	18.50	20.50 ab	13.14 ab	12.43

The presented values in the table are the mean of different levels of the studied traits. Statistical significance in each group was analysed by one-way ANOVA with multiple comparisons being made by Tukey’s test. Significant differences between level groups are indicated by letters (*p* < 0.05). PH, BrN, BN, SW and SI are abbreviated for plant height, branch number, boll number, seed set weight (g), seed index (g), respectively

**Fig. 4:**
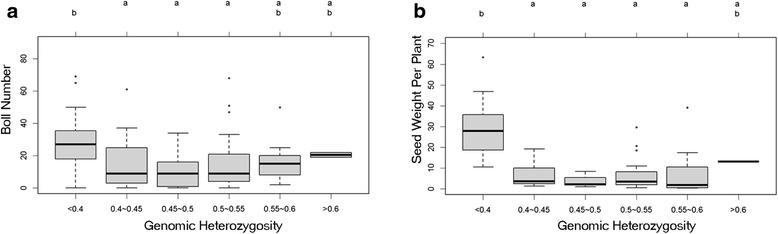
Boxplot of plant traits across groups of genomic heterozygosity for boll number (**a**) and seed set weight (**b**). Multiple group comparisons were performed using one-way ANOVA followed by Tukey’s multiple comparison tests. The bottoms and tops of the boxes represent 25 and 75 %, respectively; the bands near the middle indicate the median. The ends of the whiskers represent 10 and 90 %. The letters above the boxes indicate significant differences between the groups. Groups that do not share a letter differ significantly (*p* < 0.05)

The results from the pairwise comparisons between BN and SW in the (E3) F_2_ population are shown in Table 5 and Fig. 4. Significant differences for BN and SW were detected between level 1 and levels 2, 3, 4, as well as between level 1 and levels 2, 3, 4, 5. No significant differences were detected among the other pairwise comparisons.

According to the definition of MHI, higher MHI corresponds to higher levels of individual genomic heterozygosity, when MHI values are less than 0.5. Through comparative analysis of BN and SW at different levels in the (E3) F_2_ population, we conclude that increased genomic heterozygosity indicates lower BN and SW values when MHI is less than 0.5. These results are consistent with our Pearson’s correlation analysis, reconfirming the negative correlations between MHI and BN and between MHI and SW in the (E3) F_2_ population. As such, hybrid breakdown in BN and SW exhibited positive correlations with genomic heterozygosity. However, this was not valid in the (3E) F_2_ population.

## Discussion

### A novel approach for assessing genomic heterozygosity

Heterozygosity–fitness correlations have been used to study the relationship between genomic heterozygosity and fitness-related traits at an individual level in natural hybrid populations of a variety of organisms [8–12]. Nonetheless, associations of marker loci have been ignored in previous studies of genomic heterozygosity, which could give rise to an estimate bias if such associations are true. As an extreme example, calculated genomic heterozygosity only represents chromosome heterozygosity, in cases where all markers are located on the same chromosome.

In the current study, the loci for MHI calculating were randomly selected from the 2316 genome-wide loci cotton interspecific genetic map [34], the correlation between paired loci were checked by LD analysis, independent assortment between the loci must be validated. Genome-wide LD patterns in hybrid populations have been studied in a few organisms [44–46]. These studies suggested that LD analysis can be used to estimate correlation among marker loci in hybrid populations.

In our study, a total of 83 common loci were determined through marker-checking, which were subsequently used to assess genomic heterozygosity. To the best of our knowledge, this is an improved approach for assessing genomic heterozygosity, which could be applicable to many species that are currently the focus of heterozygosity–fitness correlation research in evolution and ecology. It could also provide new insights for assessing general genome-wide heterozygosity.

### Hybrid breakdown in interspecific reciprocal F_2_ cotton populations

Previous works have documented hybrid breakdown in the F_2_ and later generations of interspecific hybrids between *G. hirsutum* and *G. barbadense* [33, 47–49]. For example, Jiang et al. [47] reported that multilocus epistatic interactions affected gene transmission in an interspecific population of polyploid *Gossypium*. However, such studies have not focused on the effect of hetero-species cytoplasm on hybrid breakdown in cotton interspecific reciprocal populations. In the current study, we assessed the effect of hetero-species cytoplasm by comparing plant trait performance in cotton interspecific reciprocal F_2_ populations. The cytoplasmic environment had significant effects on PH and SI, and this study therefore suggested that the cytoplasmic environment might play an important role in hybrid breakdown in cotton interspecific reciprocal F_2_ populations.

### Effect of genomic heterozygosity on hybrid breakdown

Relationships between individual genomic-wide general heterozygosity and phenotypic traits have been studied both in both animals and plants [8, 9, 24, 29, 50]. According to previous studies, the relationships between individual genome-wide general heterozygosity and traits were very sophisticated and might differ with respect to sampling species and assessment procedure. For example, significant positive effects between standardized multilocus heterozygosity as calculated by neutral loci and adult survival were found in blue tits (*Cyanistes caeruleus*) [11]. Abrahamsson et al. [10] found that standardized multilocus heterozygosity in mother trees had a significant negative effect on mean offspring height in an inbred Scots pine population. Savolainen and Hedrick [8] had reported that no evidence of an association was found between vegetative and reproductive fitness-related traits and heterozygosity in *P. sylvestris*.

The majority of current studies on heterozygosity–fitness correlations have focused on natural populations in hybrid zones. In the current study, we assessed relationships between genomic heterozygosity and hybrid traits in two artificial hybrid populations derived from a reciprocal cross between *G. hirsutum* and *G. barbadense*. In the (3E) F_2_ population, no correlations were observed between the measured plant traits and genomic heterozygosity, indicating that hybrid breakdown may be not linked with genomic heterozygosity. On the other hand, correlations were only observed between SI, BN and genomic heterozygosity in the (E3) F_2_ population. These findings illustrated that cytoplasms from different species might significantly impact such relationships.

Our results also indicated that hybrid performance in the *G. barbadense* cytoplasmic background might have no correlation with genomic heterozygosity, regardless of whether vegetative traits or reproductive traits were being assessed; however, in the *G. hirsutum* cytoplasmic background, the reproductive traits SI and BN were associated with genomic heterozygosity. These results suggested that the *G. barbadense* cytoplasm background could exhibit better compatibility compared with that of *G. hirsutum*. Our results may therefore offer new insights into hybrid breakdown in allotetraploid cotton interspecific hybrids and may provide a fresh perspective on interspecific hybridization for the genetic improvement of cotton.

## Conclusions

To investigate the correlations between genomic heterozygosity and vegetative and reproductive traits in allotetraploid cotton, two reciprocal F_2_ populations were developed using *G. hirsutum* cv. Emian 22 and *G. barbadense* acc. 3–79 as parents. A total of 125 SSR markers were evaluated by marker pair correlations using linkage disequilibrium analysis, and 83 common loci were used to assess the extent of genomic heterozygosity. The vegetative traits (PH and BrN) and reproductive traits (BN, SW and SI) were investigated in the in allotetraploid cotton interspecific populations, and hybrid breakdown was found extensively in the two interspecific F_2_ populations particularly on the reproductive traits because of the infertility and the bare seeds. The only relationships between hybrid breakdown and heterozygosity was observed for SI and BN in the (E3) F_2_ population. The maternal cytoplasmic environment may have a significant effect on genomic heterozygosity and on correlations between heterozygosity and reproductive traits.

## Ethics (and consent to participate)

Not applicable.

## Consent to publish

Not applicable.

## Availability of data and materials

All relevant data are available within the manuscript and its additional files.

## Additional files

Additional file 1: Table S1. Markers’ information in this research. (XLS 42 kb) Additional file 2: Table S2. MHIs in (3E) F_2_ population. (XLS 26 kb) Additional file 3: Table S3. MHIs in (E3) F_2_ population. (XLS 14 kb) Additional file 4: Table S4. The QTL for studied traits searched from the cotton QTL Database. (XLS 25 kb)

## Abbreviations

ANOVA: analysis of variance
ATP: adenosine triphosphate
BDM incompatibilities: Bateson–Dobzhansky–Muller (BDM) incompatibilities
BN: boll number
BrN: branch number
E22: Emian 22
LD: linkage disequilibrium
MHI: molecular hybrid index
PH: plant height
QTL: quantitative trait loci
SI: seed index
SNPs: single-nucleotide polymorphisms
SSRs: simple sequence repeats
SW: seed set weight
